# A Novel Cancer Testis Antigen, A-Kinase Anchor Protein 4 (AKAP4) Is a Potential Biomarker for Breast Cancer

**DOI:** 10.1371/journal.pone.0057095

**Published:** 2013-02-22

**Authors:** Shikha Saini, Nirmala Jagadish, Anju Gupta, Amar Bhatnagar, Anil Suri

**Affiliations:** 1 Cancer Microarray, Genes and Proteins Laboratory, National Institute of Immunology, Aruna Asaf Ali Marg, New Delhi, India; 2 NMC Imaging and Diagnostic Centre, Vidyasagar Institute of Mental Health and Neuro-Sciences, New Delhi, India; 3 Department of Cancer Surgery, Safdarjung Hospital and Vardhman Mahavir Medical College, New Delhi, India; Dartmouth, United States of America

## Abstract

**Background:**

Breast cancer is the second leading cause of cancer related deaths in women worldwide. Reports about the early diagnosis of breast cancer are suggestive of an improved clinical outcome and overall survival rate in cancer patients. Therefore, cancer screening biomarker for early detection and diagnosis is urgently required for timely treatment and better cancer management. In this context, we investigated an association of cancer testis antigen, A-Kinase anchor protein 4 (AKAP4) with breast carcinoma.

**Methodology/Findings:**

We first compared the AKAP4 gene and protein expression in four breast cancer cells (MCF7, MDA-MB-231, SK-BR3 and BT474) and normal human mammary epithelial cells. In addition, 91 clinical specimens of breast cancer patients of various histotypes including ductal carcinoma in situ, infiltrating ductal carcinoma and infiltrating lobular carcinoma and 83 available matched adjacent non-cancerous tissues were examined for AKAP4 gene and protein expression by employing *in situ* RNA hybridization and immunohistochemistry respectively. Humoral response against AKAP4 was also investigated in breast cancer patients employing ELISA. Our *in vitro* studies in all breast cancer cells revealed AKAP4 gene and protein expression whereas, normal human mammary epithelial cells failed to show any expression. Using *in situ* RNA hybridization and immunohistochemistry, 85% (77/91) tissue specimens irrespective of histotypes, stages and grades of breast cancer clinical specimens revealed AKAP4 gene and protein expression. However, matched adjacent non-cancerous tissues failed to display any AKAP4 gene and protein expression. Furthermore, humoral response was observed in 79% (72/91) of total breast cancer patients. Interestingly, we observed that 94% (72/77) of breast cancer patients found positive for AKAP4 protein expression generated humoral response against AKAP4 protein.

**Conclusions:**

Collectively, our data suggests that AKAP4 may be used as serum based diagnostic test for an early detection and diagnosis of breast cancer and may be a potential target for immunotherapeutic use.

## Introduction

Breast cancer is the most commonly diagnosed cancer and is the second leading cause of cancer related deaths in women worldwide [1]. Recent published breast cancer statistics indicated that estimated 226,870 new cases of invasive breast cancer are expected to occur among women in 2012 [1]. The mortality rate in developing countries is even higher because of limited medical infrastructure and awareness [1]. Further, reports on breast cancer have shown that early diagnosis directly contributes to improved clinical outcome and over-all survival in breast cancer patients [2]. This substantiates the necessity to explore a novel tissue or serum based biomarker which can help in early detection and diagnosis of breast cancer for better cancer management.

Multiple tumor biomarkers have been reported in breast cancer including carcinoembryonic antigen (CEA), mucin 1 (MUC1) and CA 15-3 [3]. However, none of these biomarkers have been implicated in clinical practice because of false-positive rate in normal populations, and low diagnostic sensitivity and specificity [3]. The gold standard detection method for breast cancer used in routine clinical practice is mammography. However, mammography screening has limitations in its inability to detect early stage cancers and false-positives (8–10%) diagnosis in cases of dense breast tissues or calcifications [4]. Moreover, early stages in breast cancer are often asymptomatic leading to the delayed diagnosis when the effective treatment modalities options are very few.

A unique class of antigens designated as Cancer testis (CT) antigens are considered to be clinically important as biomarkers and therapeutic targets because of their expression in various cancers and high immunogenicity profiles [5]. Our previous studies have demonstrated the expression of a novel CT antigen, Sperm associated antigen 9 (SPAG9) in various cancers and showed its association with cellular proliferation, migration and invasion [6]–[13]. Further, our studies demonstrated SPAG9 expression in 88% of breast cancer patients. In addition, humoral immune response against SPAG9 was also observed in 80% of early stages and low-grade breast cancer patients [8]. Therefore, these CT antigens might serve as tumor specific biomarkers and immunotherapeutic targets.

Earlier, we reported a novel testis specific gene designated as *AKAP4* having exclusive expression in testis and not in any other normal tissues tested [14]. Moreover, AKAP4 expression was recently validated by employing microarray gene expression analysis that revealed restricted AKAP4 expression only in testis and in various cancer cells [15]. AKAP4 functions as a scaffolding protein and tethers cAMP dependent Protein Kinase A (PKA) [16] where PKA has been proposed to be involved in majority of human tumors and malignant properties including cell proliferation, angiogenesis, and chemoresistance [17]–[20]. In this regard, AKAP4 was recently shown to be associated with multiple myeloma [21], prostate cancer [22] and lung cancer [23]. The involvement of AKAP4 is suggestive of its pivotal role in tumorigenesis and holds promise to serve as a biomarker for better clinical management of cancer patients. Therefore, this investigation was undertaken to assess the association of AKAP4 with various clinical parameters in order to develop tissue or serum based early detection biomarker for better cancer treatment modalities in breast cancer patients.

## Materials and Methods

### Cell Lines

Human normal mammary epithelial cells, three breast adenocarcinoma cell lines (MCF7, MDA-MB-231 and SK-BR3), and a ductal carcinoma cell line (BT474) were used in this study. Human normal mammary epithelial cells were purchased and maintained according to manufacturer’s directions (Gibco, Life Technologies Corporation, Carlbad, CA). MCF 7 and MDA-MB-231 were purchased from American Type Culture Collection (ATCC, Manassas, VA). BT474 and SK-BR3 cell lines were also procured from ATCC by Prof. Susan E. Kane (Division of Tumor Cell Biology, Beckman Research Institute of City of Hope, Duarte, CA) and were kindly gifted for the experiments [24]. MCF7 and MDA-MB-231 cell lines were maintained in Dulbecco modified Eagle medium, BT474 in RPMI-1640 and SK-BR3 in McCoy’s 5A media. Complete media was prepared by adding 10% fetal calf serum, 50 mg/mL gentamycin, and 100 mg/mL streptomycin (Invitrogen, Carlsbad, CA) and were maintained at 37°C in a humidified atmosphere with 5% CO2.

### Patient’s Tissue Specimens and Ethics Statement

A total of 91 breast tumor tissue including 4 Ductal Carcinoma in situ (DCIS), 83 Infiltrating Ductal carcinoma (IDC) and 4 Infiltrating Lobular Carcinoma (ILC) and 83 matched available adjacent non-cancerous tissue (ANCT) specimens were collected during routine surgical procedure from All India Institute of Medical Sciences after obtaining the informed written consent form from patients and surgeons. The approval for conducting the research was obtained from the institutional human ethical committee of All India Institute of Medical Sciences, New Delhi and National institute of Immunology. Tumor tissue samples and ANCT specimens were fixed in 5% buffered formaldehyde and processed for immunohistochemistry. Tissue specimens were also fixed in RNase free fixative for *in situ* RNA hybridization studies. In addition, sera samples of the cancer patients and healthy donors (45 females and 19 males) were also collected and stored at −80°C until use for determining humoral response against AKAP4.

### *AKAP4* Gene Cloning, Protein Expression and Generation of Polyclonal Antibodies in Rats

Human AKAP4 cDNA [GenBank: Y15195] encoding a reading frame of 1–140 amino acids (N terminus) was cloned in pET28b+ vector (Novagen, Madison, WI) encoding a His6-tagged fusion protein. The plasmid was further transformed in *E.coli* BL21 (DE3) for protein expression using standard protocols. The protein expression was induced by adding 1 mM Isopropyl β-D-1thiogalactopyranoside (IPTG) for 4 h during log phase. The expressed His6–tagged protein was further purified by Ni-NTA based affinity chromatography and sequence was confirmed by microsequencing with tandem mass spectrometry. Polyclonal antibodies to recombinant AKAP4 were raised using CFA as an adjuvant in rats. Further, rat serum IgG was isolated using Nab Protein G Spin Chromatography kit (Pierce, Rockford, IL) according to the manufacturer’s protocol and used as anti-AKAP4 antibody for all our experiments. To determine the specificity of anti-AKAP4 antibody, neutralization experiment was carried out, wherein, anti-AKAP4 antibody or patient’s sera at dilution of 1∶10 was pre-incubated with 15 µg/ml of recombinant AKAP4 protein and then used for probing recombinant purified protein by employing western blotting. Furthermore, non-specific binding of anti-AKAP4 antibody with other *E.coli* BL21 (DE3) proteins was ruled out by Western blot analysis. Control IgG antibody was also purified in a similar manner from preimmunized rat’s sera and used as a negative control in all subsequent experiments.

### Reverse-Transcriptase-PCR Analysis

Total RNA was extracted from various cancer cell lines using the TRI reagent (Ambion, Inc., Austin, TX) according to the manufacturer’s protocol. RNA was dissolved in DEPC-treated water and concentration was determined. cDNA was synthesized from RNA using Accuscript Highfidelity cDNA synthesis kits (Stratagene, La Jolla, CA). RT-PCR was done using cDNA as a template and following AKAP4 primers (forward 5′-TGATACTACAATGATGTCTGATGAT-3′ and reverse 5′GGAACTAGCAGCATCCTTGTAATCTTTATC -3′). Subsequently, 40 amplification cycles [1 cycle of denaturation at 94°C for 2 min, 40 cycles: denaturation at 94°C for 45 s; annealing at 45°C for 45 s; extension at 72°C for 45 sec; and a final elongation cycle at 72°C for 7 min] were carried out for each sample. The PCR products were analyzed on 2% agarose gels and photographed under UV light. RT-PCR for β-actin mRNA expression was done as an internal control. The sequences for forward and reverse primers of *β-actin* were 5′-ATCTGGCACCACACCTTCTACAATGAGCTGCG-3′ and 5′- CGTCATACTCCTGCTTGCTGATCCACATCTGC-3′. Subsequently, the PCR products was sub-cloned into TOPO vector using TOPO kit (Invitrogen/Life Technologies, Carlsbad, CA) and the nucleotide sequence was confirmed by automated DNA sequencing.

### Cell Specific Localization of *AKAP4* Transcript by *in situ* RNA Hybridization

The 3′ end of cDNA fragment of coding region of published human AKAP4 (from 2, 461–2,710 bp) was sub-cloned in pBluescript SK (+/−) as described earlier [14]. Dioxigenin (DIG)-11-UTP conjugated riboprobes were prepared using T7 or T3 RNA polymerase for the antisense or sense *in vitro* transcript using DIG RNA labeling kit (Roche Diagnosatics GmbH, Mannheim, Germany). For detection of *AKAP4* transcript in breast cancer, serial tissue sections were processed according to the manufacturer’s instructions (DIG Nucleic Acid Detection Kit, Roche Diagnosatics GmbH, Mannheim, Germany) [14].

### Western Blotting

AKAP4 protein expression was assessed by Western blotting in all four breast cancer cell lines. Briefly, 40 µg cell lysate were subjected to 10% SDS-PAGE and transferred to polyvinylidene fluoride (PVDF) membrane. Membrane was blocked with 3% non-fat skimmed milk for 45 min at room temperature and probed with rat polyclonal anti-AKAP4 antibodies at 4°C for overnight followed by secondary antibody incubation of horsereddish-peroxidase conjugated anti-rat IgG (Jackson ImmunoResearch Laboratories, West Grove, PA) for 2 h at room temperature. Immunoreactive bands were detected by enhanced chemiluminescence (ECL) using immobilon western substrate (Millipore Corporation, Billerica, MA).

### Indirect Immunofluorescence and Flow-cytometric Analysis

Cancer cells were grown on cover slips and fixed in 3% paraformaldehyde. The fixed cells were permeabilized in 0.5% igepal (Sigma-Aldrich, St. Louis, MA) for 10 min at room temperature and blocked in 1% fetal bovine serum for 10 min at room temperature. Cells were incubated with rat polyclonal anti-AKAP4 antibody in humid chamber for overnight at 4°C, followed by incubation with fluorescein isothiocyanate (FITC) conjugated anti-rat IgG antibody (Jackson ImmunoResearch Laboratories, West Grove, PA) for 2 h at room temperature. Nuclei of the cells were stained using 4′, 6-diamidino-2-phenylindole (DAPI) (Sigma-Aldrich, St. Louis, MA) and analyzed under Nikon Eclipse E 400 microscope (Nikon, Fukok, Japan). For surface localization of AKAP4 protein, live cells were probed with rat polyclonal antiAKAP4 antibody for 2 h at 4°C, followed by 1 h incubation with FITC conjugated anti-rat IgG antibody (Jackson ImmunoResearch Laboratories, West Grove, PA). After washing with filtered phosphate buffered saline (PBS), cells were fixed in 0.4% paraformaldehyde for 10 min at room temperature. Data was acquired and analyzed using cell quest software in BD FACS Calibur (Becton Dickinson, San Jose, CA).

### Immunohistochemical Analysis

Breast cancer tissue specimens were analyzed for localization of AKAP4 protein using rat polyclonal anti-AKAP4 antibody or control IgG as described earlier [8]. Briefly, paraffin-embedded tissue sections were blocked with 5% normal goat serum for 30 min after deparaffinization, rehydration and endogenous peroxidase removal. Specimens were incubated with polyclonal anti-AKAP4 antibody or control IgG at 4°C for overnight in humid chamber. Subsequently, the sections were incubated for 1 h at room temperature with secondary antibody of horseradish peroxidase-conjugated goat anti-rat IgG (Jackson ImmunoResearch Laboratories, West Grove, PA). Serial sections were processed for proliferation marker (ki67) in all three histological grades. Monoclonal mouse anti-Ki67 anitbody (Dako, Carpinteria, CA) was used to probe Ki67 protein, followed by incubation with horseradish peroxidase-conjugated goat anti-mouse IgG (Jackson ImmunoResearch Laboratories, West Grove, PA). Reactivity in the tissue specimens was visualized using chromogen 0.05% 3, 3′-diaminobenzidine (Sigma-Aldrich, St. Louis, MA). To determine the specificity of anti-AKAP4 antibody, neutralization experiment was carried out, wherein, anti-AKAP4 antibody at dilution of 1∶10 was pre-incubated with 15 µg/ml of recombinant AKAP4 protein and then used for probing endogenous AKAP4 protein in IDC tissue specimens by employing IHC. AKAP4 Immunoreactivity score (IRS) was calculated as a percentage of AKAP4 expression positive cells by counting >500 cells from 5 random fields of each section for each specimen by the senior pathologist. We considered a distinct positive immunoreactivity in a specimen showing >10% of cancer cells stained for AKAP4 protein.

### Circulating Anti-AKAP4 Antibodies Detection in Breast Cancer Patients

Recombinant AKAP4 purified protein at the concentration of 4 µg/ml in coating buffer (15 mmol/L Na2CO3, 35 mmol/L NaHCO3, pH 9.4) was coated in 96-well plates (Nunc, Roskilde, Denmark) for overnight at 4°C. Non-specific sites were blocked with 3% non-fat skimmed milk for 1 h at room temperature and incubated with breast cancer patients or healthy normal’s sera in blocking solution for 2 h at room temperature. Plates were washed with PBS containing 0.5% Tween 20 (PBST) and incubated with horsereddish-peroxidase conjugated anti-human IgG (Jackson Immunoresearch Laboratories, West Grove, PA) for 1 h at room temperature. Absorbance was observed colorimetrically at 492 nm by using o-Phenylenediamine dihydrochloride as a substrate. All breast cancer patients and healthy donor samples were tested in duplicates and mean was used for analysis. The inter-assay and intra-assay coefficients of variation were calculated from three independent experiments.

Humoral response was further confirmed by Western blotting. 0.5 µg of purified recombinant AKAP4 protein was resolved on 12% SDS-PAGE and transferred. Membrane was incubated with sera of cancer patients and healthy donors (1∶400) for overnight at 4°C and subsequently with secondary antibody. To validate the antibodies generated against AKAP4 in cancer patients, patient’s sera were pre-incubated with 15 µg/ml of recombinant AKAP4 protein for 2 h at 4°C. The pre-incubated patient’s sera were subsequently used as a primary antibody and horsereddish-peroxidase conjugated anti-human IgG (Jackson Immunoresearch Laboratories, West Grove, PA) as a secondary antibody for neutralization experiments. Immunoreactivity was detected by standard ECL procedures.

### Statistical Analysis

Data are expressed as mean±standard error of at least three independent experiments. All statistical analysis was performed by employing SPSS software version 19 (SPSS, Chicago, IL). Pearson’s Chi-square test, Mann Whitney U-test and Kruskal Wallis test were used to analyze the AKAP4 expression and humoral response in various clinical subgroups including various stages, grades and histotypes. P value of <0.05 was considered statistically significant.

## Results

### AKAP4 Gene and Protein is Expressed in Breast Cancer Cell Lines

**Figure 1:**
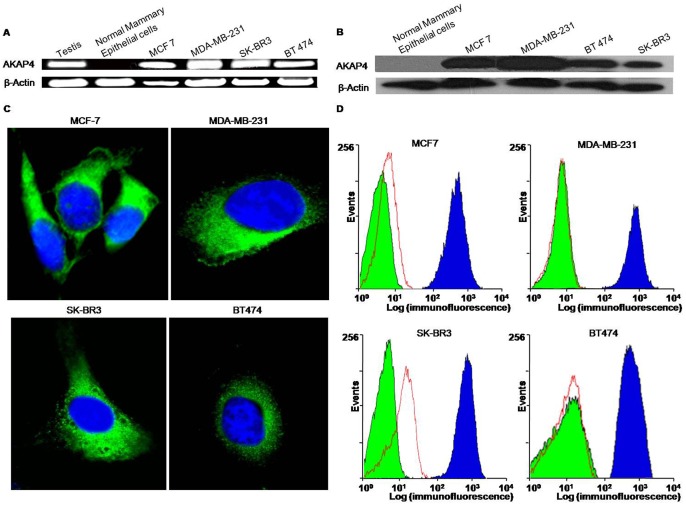
AKAP4 expression and localization in breast cancer cell lines. A, Reverse-transcriptase PCR analysis showing *AKAP4* gene expression in testis, all breast cancer cell lines, MCF7, MDA-MB 231, SK-BR3 and BT474 but not in normal human mammary epithelial cells. B, AKAP4 protein expression was detected by employing Western blotting. C, Cells were fixed, permeabilized and processed for indirect immunofluorescence studies which revealed cytoplasmic localization (green color) of AKAP4 protein. Nuclei were stained blue using DAPI. D, Flow cytometric analysis of live cells demonstrating surface expression of AKAP4 protein in all breast cancer cell lines, MCF7, MDA­MB-231, SK-BR3 and BT474. The surface expression of AKAP4 protein (blue histogram) is depicted by the shift of fluorescence on X-axis with respect to unstained cells (green histogram) and control IgG stained cells (red histogram). Analysis was done using BD cell quest software.

AKAP4 gene expression was investigated in human normal mammary epithelial cells and breast cancer cells of two different origins namely adenocarcinoma (MCF7, MDA-MB-231 and SK-BR3), and ductal carcinoma (BT474). In order to avoid the genomic DNA amplification, AKAP4 primers were designed from overlapping exons (exon1/2 forward and exon 4/3 reverse primers) and were used for reverse-transcriptase polymerase chain reaction (RT-PCR). Our results revealed *AKAP4* gene expression in all four breast cancer cell lines (MCF7, MDA-MB-231, SK-BR3 and BT474), but not in normal mammary epithelial cells (Figure 1A). The amplicon was subsequently cloned in TOPO vector and sequenced which revealed no mutation in AKAP4 nucleotide sequence as reported in testis [14]. *AKAP4* gene expression was validated for endogenous AKAP4 protein expression in all breast cancer cells by using Western blotting. Polyclonal anti-AKAP4 antibody raised in rats was used to probe the AKAP4 protein in breast cancer cell lysates. As shown in Figure 1B, AKAP4 protein expression was detected in all the breast cancer cell lines but not in normal breast epithelial cells. β-actin was used as an internal loading control for Western blotting experiments.

We further examined the localization of AKAP4 protein in breast cancer cells by indirect immunofluorescence assay and flow cytometric analysis. For indirect immunofluorescence, breast cancer cells were fixed, permeabilized and probed with anti-AKAP4 antibodies. All breast cancer cell lines revealed cytoplasmic localization of AKAP4 protein (Figure 1C). Subsequently, we also investigated the AKAP4 surface expression in live breast cancer cells by flow cytometry which revealed a distinct shift of fluorescence on X-axis (blue histogram) indicating AKAP4 protein localization on the surface of the cells as shown in Figure 1D. In contrast, cells that were probed with control IgG (Figure 1D, red histogram) showed no displacement with respect to unstained cells (Figure 1D, green histogram). The surface localization of AKAP4 protein suggests that it may be a potential target candidate for therapeutic use in breast cancer patients.

### *AKAP4* Gene is Expressed in Breast Cancer Tissue Specimens

**Figure 2:**
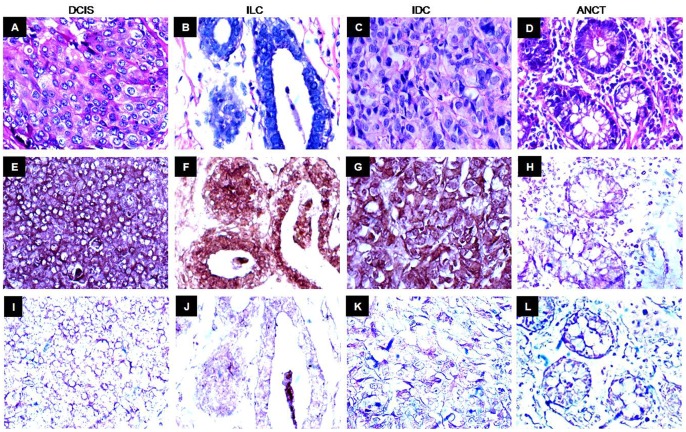
Gene expression analysis of *AKAP4* in breast cancer tissue specimens. Serial tissue sections of different histotypes (DCIS, IDC ILC) and matched ANCT specimens were processed for in situ RNA hybridization and probed with DIG-labeled anti-sense (complementary sequence as that of AKAP4 transcript) and sense riboprobe (same sequence as that of AKAP4 transcript). Panels A–D, H & E staining showing the cytostructure of representative microphotograph of DCIS, ILC and IDC and ANCT tissue specimens respectively. Panels E–H, representative microphotographs probed with anti-sense riboprobe showing AKAP4 gene expression, depicted by chocolate brown color in all histotypes of breast cancer. AKAP4 transcript was detected in 100% DCIS (4/4), 83% IDC (69/83) and 100% ILC (4/4) tissue specimens. Panels I–L, sense riboprobe failed to display reactivity in any tissue section. It is noteworthy that no reactivity was observed in matched ANCT probed with anti­sense riboprobe. (Original magnification 400; Objective - X40).

AKAP4 gene expression was investigated in breast cancer patient’s tissue specimens by employing in situ RNA hybridization. Our results revealed hybridization of anti-sense AKAP4 riboprobe depicted by chocolate brown color in cells expressing AKAP4 gene (Figure 2). As expected, sense riboprobe, having the same sequence as that of endogenous AKAP4 mRNA failed to show hybridization and resulted in no reactivity. AKAP4 gene expression was detected in 85% (77/91) of breast cancer patients. Among the various histotypes, AKAP4 gene expression was detected in 100% DCIS (4/4), 83% IDC (69/83) and 100 ILC (4/4) specimens (Figure 2, Table 1). Although the number of specimens was less in DCIS and ILC, however, both these histotypes revealed AKAP4 gene expression in 100% of specimens tested. Interestingly, the more prevalent breast cancer, IDC also revealed AKAP4 gene expression in majority of IDC cancer patient’s specimens under investigation. No AKAP4 gene expression was detected in available matched (ANCT) specimens. The AKAP4 mRNA expression in all clinicopathological characteristics including stages and pathologic grades of investigated specimens are shown in Table 1.

**Table 1:** Clinopathologic features, AKAP4 expression and humoral response in breast cancer.

Clinicopathologic features	AKAP4 gene/protein expression	AKAP4 humoral response
Total breast cancer tissues specimens	77/91 (85%)	72/91(79%)
Adjacent normal cancerous tissues	0/83 (0%)	–
**Histotypes**		
DCIS	4/4 (100%)	4/4 (100%)
IDC	69/83 (83%)	64/83 (77%)
ILC	4/4 (100%)	4/4 (100%)
**Clinical Stages**		
Early stage (stage I+stage II)	45/52 (87%)	42/52 (81%)
Stage I	1/1 (100%)	1/1 (100%)
Stage II	44/51 (86%)	41/51 (80%)
Late stage (stage III+stage IV)	32/39 (82%)	30/39 (77%)
Stage III	31/38 (82%)	29/38 (76%)
Stage IV	1/1 (100%)	1/1 (100%)
**Histopathological Grades**		
Grade 1	38/47 (81%)	36/47 (77%)
Grade 2	32/36 (89%)	29/36 (81%)
Grade 3	7/8 (88%)	7/8 (88%)

DCIS, Ductal Carcinoma in situ; IDC, Infiltrating Ductal Carcinoma; ILC, Infiltrating Lobular Carcinoma.

### AKAP4 Protein is Expressed in Breast Cancer Tissue Specimens

**Figure 3:**
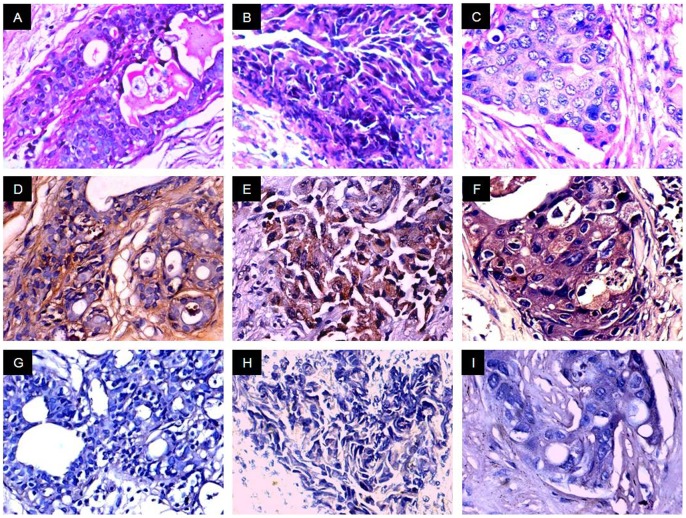
AKAP4 protein expression immunohistochemical studies in DCIS tissue specimens. Panels A–C, H & E stained representative microphotographs showing cytostructure of DCIS tissue sections. Panels D–F, serial tissue sections of three representative specimens probed with anti-AKAP4 antibody showed distinct cytoplamic localization of AKAP4 protein (brown color). Panels G–I, serial sections probed with control IgG failed to show any immunoreactivity. (Original magnification 400; Objective - X40).

**Figure 4:**
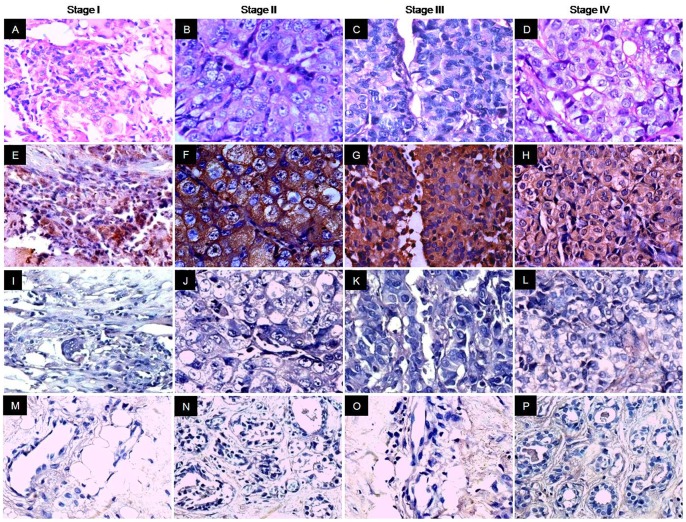
Immunohistochemical analysis of AKAP4 protein expression in different stages of IDC tissue specimens. Panels A–D, H & E staining showing the histological cytostructure of representative stage I, stage II, stage III and stage IV tissue specimens respectively. Panels E–H, AKAP4 immunoreactivity was observed in the cytoplasm in all stages (brown color). Overall, 100% stage I (1/1), 86% stage II (44/51), 82% stage III (31/38) and 100% stage IV (100%) clinical specimens were found positive for AKAP4 protein expression. Panels I–L, no immunoreactivity was detected with control IgG probed specimens. Panels M- P, AKAP4 protein was not detected in any of the matched ANCT specimens probed with anti-AKAP4 antibodies. (Original magnification 400; Objective - X40).

**Figure 5:**
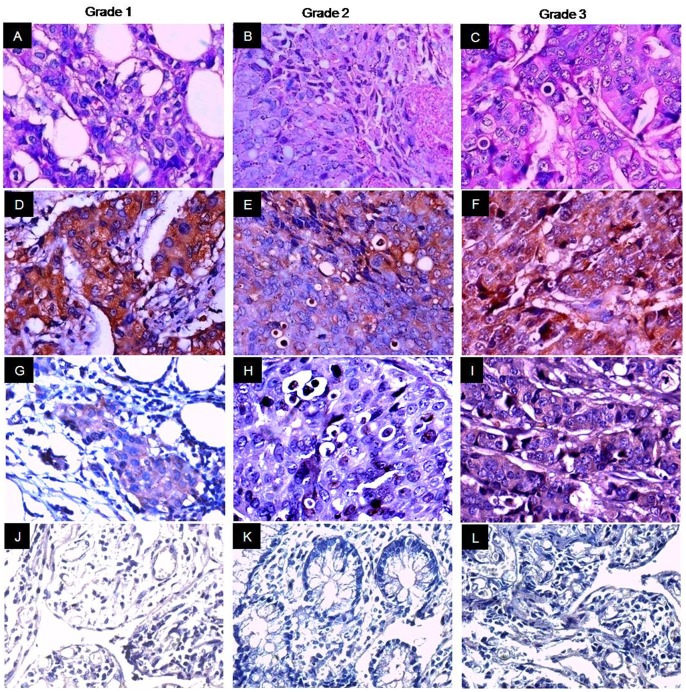
Grade-wise AKAP4 protein expression in IDC tissue specimens. Panels A–C, H & E stained histological analysis of representative microphotographs of grade 1, grade 2 and grade 3 tissue specimens respectively. Panels D–F, representative microphotograph of each grade is shown in the figure. AKAP4 protein was expressed in all grades as depicted by cytoplasmic brown immunoreactivity. AKAP4 immunoreactivity was detected in 81% grade 1 (38/47), 89% grade 2 (32/36) and 88% grade 3 (7/8) breast cancer patients probed with anti­AKAP4 antibodies. Panels G–I, Ki-67 staining was used as proliferation marker for different grades. Panels J–L, matched ANCT specimens failed to show AKAP4 reactivity probed with anti-AKAP4 antibodies. (Original magnification 400; Objective - X40).

**Figure 6:**
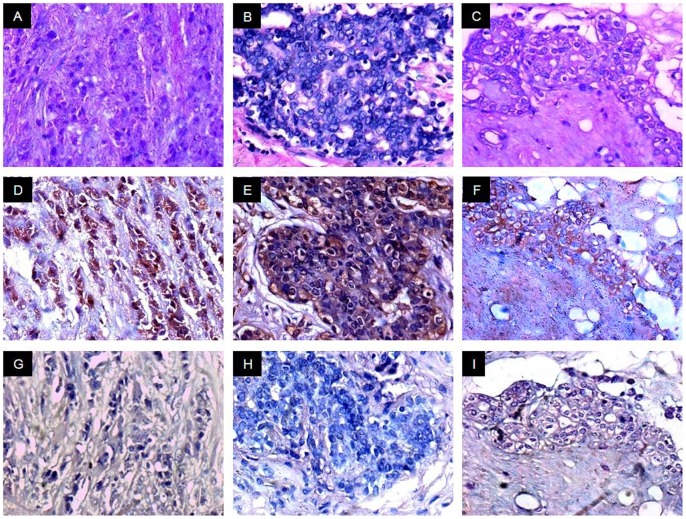
AKAP4 protein expression analyses ILC tissue specimens. Panels A–C, H & E stained representative microphotographs of ILC tissue specimens respectively. Panels D–F, three of the representative microphotographs are shown here. Distinct AKAP4 cytoplasmic immunoreactivity was detected in 100% ILC (4/4) specimens probed with anti-AKAP4 anti-antibodies. Panels G–I, serial tissue sections probed with control IgG resulted in no reactivity. (Original magnification 400; Objective - X40).

Validation of AKAP4 protein expression was carried on serial sections of breast tissue specimens used for in situ RNA hybridization by employing immunohistochemistry (IHC). Our results distinctly revealed cytoplasmic localization of AKAP4 in 85% (77/91) of breast cancer patients. It is noteworthy that we did not observe discrepancy between AKAP4 gene and protein expression in breast cancer specimens under investigation (Table 1). We analyzed AKAP4 protein expression in all histotypes using anti-AKAP4 antibodies which revealed AKAP4 protein localization in 100% DCIS (4/4) (Figure 3), 83% IDC (69/83) (Figure 4, 5) and 100% ILC (4/4) (Figure 6) patient’s specimens (Table 1) suggesting its importance for developing as a biomarker. Serial tissue sections probed with control IgG showed no immunoreactivity.

Similarly, no AKAP4 protein expression was detected in ANCT specimens indicating that AKAP4 expression was associated with cancerous tissues (P<0.001, Pearson’s Chi-square test). We further compared the AKAP4 IRS among various histotypes. The average AKAP4 IRS was 75.35±8.88 (DCIS), 61.22±8.36 (IDC) and 53.6±9.35 (ILC) [Figure S1A]. While comparing various histotypes, no significant difference in AKAP4 expression was observed by using Kruskal Wallis test (P = 0.365), as shown in Table 2.

**Table 2:** Statistical analysis of AKAP4 expression and humoral response with different clinical characteristics of breast cancer.

Statistical Analysis (P values)	Pearson’s χ^2^ test		Mann Whitney U-test		Kruskal-Wallis test	
Clinicopathologic characteristics	AKAP4 IHC IRS	AKAP4 ELISA	AKAP4 IHC IRS	AKAP4 ELISA	AKAP4 IHC IRS	AKAP4 ELISA
DCIS, IDC and ILC	0.451	0.314	–	–	0.365	0.302
Early and late stages	0.557	0.655	0.865	0.545	–	–
Grades 1, 2 and 3	0.586	0.753	–	–	0.262	0.346
Grade 1 and 2	0.318	0.664	0.187	0.183	–	–
Grade 2 and 3	0.911	0.645	0.534	0.308	–	–
Grade 1 and 3	0.652	0.490	0.216	0.974	–	–

DCIS, Ductal Carcinoma in situ; IDC, Infiltrating Ductal Carcinoma; ILC, Infiltrating Lobular Carcinoma; IRS, Immunoreactivity Score.

We subsequently assessed our findings with respect to various clinicopathological parameters including clinical stages and grades. AKAP4 protein was detected in all stages of breast cancer, as represented in Figure 4. AKAP4 protein expression was found in 87% early stages (stage I+II) and 82% late stages (stage III+IV) breast cancer patients (Table 1). The average AKAP4 IRS in early stage and late stage tissue specimens were 60.94±3.5 and 62.43±3.87 respectively (Figure S2A). It is interesting to note that AKAP4 protein was expressed in majority of early stages as well as late stages cancer patients and there was no significant difference in AKAP4 expression found between these clinical sub-groups by using Mann Whitney U-test (P = 0.865) suggesting its broad clinical utility as a biomarker (Table 2). We further extended our analysis of AKAP4 expression in different grades of breast cancer samples. AKAP4 protein was expressed in 81% grade 1 (38/47), 89% grade 2 (32/36) and 88% grade 3 (7/8) specimens as shown in Figure 5 (Table 1). The mean AKAP4 IRS was 57.96±3.18 in grade 1, 64.44±4.02 in grade 2 and 67.91±10.45 in grade 3 tissue specimens (Figure S3A). It is noteworthy that IRS analysis of AKAP4 expression was observed in higher number of patients in grade 2 (89%) and grade 3 (88%) as compared to grade 1 (81%). While comparing various grades, no significant difference in AKAP4 expression was observed by using Kruskal Wallis test (P = 0.262), as shown in Table 2. The AKAP4 protein expression in majority of cancer patients irrespective of their clinical stages and histopathological grades indicates that AKAP4 may have a potential role in disease progression. Therefore, further studies are warranted in large number of patients to validate our findings.

### Anti-AKAP4 Antibodies are Detected in Majority of Breast Cancer Patients

**Figure 7:**
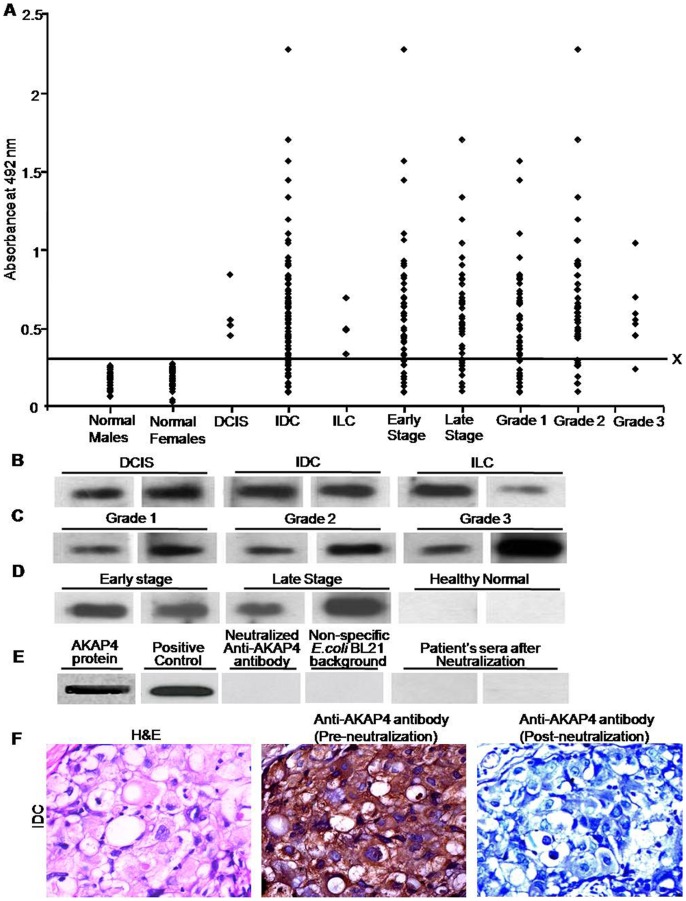
Humoral response against AKAP4 in breast cancer patients. A, To detect circulating anti-AKAP4 antibodies in cancer patient’s sera, purified recombinant AKAP4 protein was coated in 96-well plates and subjected to ELISA. Line X denotes the mean +2SD value of healthy female’s sera (absorbance value = 0.308) above which the patient’s sera was designated positive and below which the patient’s sera was designated as negative for anti-AKAP4 antibodies. All healthy males were found to be negative for anti-AKAP4 antibodies. B, Western blotting experiments were carried out by resolving purified AKAP4 recombinant protein on SDS-PAGE. Two representative cancer patient’s sera from each histotypes (DCIS, IDC and ILC) are shown depicting AKAP4 immunoreactivity. C, two representative samples showing AKAP4 immunoreactivity in different grades (grade 1, grade 2 and grade 3). D, two representative early stages and late stages sera samples showing reactivity against AKAP4. However, normal healthy sera failed to show AKAP4 immunoreactivity. E, Band 1, Coomassie stained recombinant purified AKAP4 protein; Band 2, recombinant AKAP4 protein probed with polyclonal anti-AKAP4 antibody (positive control); Band 3, recombinant AKAP4 protein probed with neutralized polyclonal anti-AKAP4 antibody (1∶10 diluted polyclonal anti-AKAP4 antibody pre-incubated with 15 µg/ml recombinant AKAP4 protein) resulted in loss of reactivity; Band 4, *E.coli* BL 21 (DE3) whole cell lysate probed with polyclonal anti-AKAP4 antibody (non-specific *E.coli* BL21 background) showed no immune-reactive band; Band 5 and 6, Representative neutralized sera samples of two cancer patients (positive for anti-AKAP4 antibodies) showed complete loss of reactivity. F. To demonstrate the specificity of anti-AKAP4 antibody in tissue specimens, serial sections were probed with anti-AKAP4 antibody and neutralized anti-AKAP4 antibody. Representative IDC specimen showing H&E staining (left), anti-AKAP4 antibody (Pre-neutralization; middle)) and neutralized anti-AKAP4 antibody (Post-neutralization; right).

We further investigated humoral response in breast cancer patients by Enzyme Linked Immuno-Sorbent Assay (ELISA) after validating AKAP4 protein expression in tissue specimens. Purified AKAP4 recombinant protein was coated in 96-well plates and used to detect anti-AKAP4 antibodies in patient’s sera. Sera from 91 breast cancer patients and from 45 healthy normal donors were subjected to ELISA for detecting anti-AKAP4 antibodies. The absorbance value of mean +2SD of healthy normal female’s sera was used as cut-off value (absorbance = 0.308) above which patient’s sera were considered positive for anti-AKAP4 antibodies. The intra-assay and inter-assay coefficients of variation were 3.6% and 5.4% respectively. Our data indicated the presence of circulating anti-AKAP4 antibodies in 79% (79/91) of the breast cancer patients. However, none of the healthy normal males showed detectable circulating anti-AKAP4 antibodies. It is important to mention that 77 patients found positive for AKAP4 protein expression, 72 patients (94%) generated humoral response against AKAP4 protein. Interestingly it is important to mention that among 69 IDC patients that were found positive for AKAP4 protein expression by IHC (69/83; Table 1), 64 IDC patients generated humoral response [93%]. Humoral response against AKAP4 was found to be strongly associated with cancer patients as compared to the healthy control donor’s samples (P<0.001, Pearson’s Chi-square test). As shown in Figure 7A, 100% DCIS (4/4), 77% IDC (64/83) and 100% ILC (4/4) patients showed humoral response against AKAP4 with mean antibody titers of 0.61±0.08, 0.73±0.04 and 0.51±0.07 respectively (Figure S1B). While comparing various histotypes, no significant difference in AKAP4 humoral response was observed by using Kruskal Wallis test (P = 0.302; Table 2). Anti-AKAP4 antibodies were found in majority of patients with early stage (81%) as well as late stage breast cancer (77%) (Table 1). The average anti-AKAP4 antibody titers in early stage and late stage specimens were 0.71±0.01 and 0.72±0.01 respectively (Figure S2B). However, among both these groups there was no significant difference in mean anti-AKAP4 antibody titers (P = 0.545, Mann Whitney U-test). Similarly, in different grades, anti-AKAP4 antibodies were detected in majority of grade 1 (77%), grade 2 (81%) and grade 3 (88%) cancer patients with mean titers of 0.66±0.05, 0.80±0.08, and 0.63±0.08 respectively (Figure S3B). While comparing various grades, no significant difference in AKAP4 humoral response was observed by using Kruskal Wallis test (P = 0.346; Table 2). Circulating antibodies against AKAP4 were found in majority of patients irrespective of their clinical stages and histological grades. Moreover, detection of humoral response against AKAP4 represents a better and minimal invasive method of diagnosis from the sera of cancer patients.

ELISA findings were subsequently confirmed by subjecting purified AKAP4 recombinant protein to sodium dodecyl sulphate - polyacrylamide gel electrophoresis (SDS-PAGE) and Western blotting experiments. All the cancer patients found positive for ELISA revealed a strong reactivity in patient’s sera of all histotypes (Figure 7B), grades (Figure 7C) and stages (Figure 7D). It is important to mention that all sera samples were preincubated with *E.coli* BL21 (DE3) cell lysate in order to remove any non-specific binding. As shown in Figure 7E, probing purified recombinant AKAP4 protein with polyclonal anti-AKAP4 antibody resulted in specific AKAP4 immunoreactive band. Interestingly, in neutralization experiment sera preincubated with 15 µg/ml of recombinant AKAP4 protein, resulted in complete loss of reactivity with AKAP4 protein. Additionally, no immunoreactivity was observed by probing *E.coli* BL21 (DE3) whole cell lysate which ensured that polyclonal anti-AKAP4 antibody was specific against AKAP4 protein. Further, to confirm the reactivity of circulating antibodies in patient’s sera against recombinant AKAP4 protein in western blotting experiment, neutralization experiments were carried out by preincubating diluted patient’s sera with 15 µg/ml of recombinant AKAP4 protein which resulted in complete loss of reactivity in immunoblotting. Similarly, to validate the specific immunoreactivity of anti-AKAP4 antibody with endogenous AKAP4 protein in cancer specimens, polyclonal anti-AKAP4 antibody was preincubated with 15 µg/ml of recombinant AKAP4 protein and used on serial tissue sections of IDC which resulted in complete loss of immunoreactivity (Figure 7F).

We further sub-divided our clinical samples in three groups based on AKAP4 IRS: 10–25%, 25–50% and 50–100% to assess the correlation of AKAP4 protein expression and humoral response. Interestingly, high anti-AKAP4 antibody titers in the patient’s sera indicated high tumor burden. Although, high titers of anti-AKAP4 antibody (>0.75) were clearly indicative of high AKAP4 protein expression (>25% AKAP4 IRS), however vice-versa did not hold true for all patients. This can be explained on the basis of heterogeneity in MHC genetics of cancer patients. Moreover, due to the heterogeneity observed in breast cancer tissues, some of the patients did not generate humoral response against AKAP4.

## Discussion

Breast cancer is the second leading cause of cancer related deaths in women worldwide with increasing incidence and mortality rate in developing countries [1]. This is more so because of lack of awareness and proper medical infrastructure to treat cancer patients [1]. In addition, it is estimated that between 15 and 25% of women with early stage breast cancer are not diagnosed by conventional mammography procedures [4]. Moreover, tumor markers presently used in early detection of breast cancer such as CA-125 and CEA lack clinical efficacy and utility [3]. Therefore, identification and characterization of new biomarkers are urgently needed for early detection and diagnosis of breast cancer for better treatment modalities and cancer management.

Recently, CT antigens have been reported in various malignancies that have restricted expression in germ cell differentiation in testis [5]. Since, testis is an immune privileged organ, where HLA molecules are not expressed, CT antigens are considered as potential biomarkers and antigen targets for immunotherapy. Unlike chemotherapeutic regimens which kill healthy dividing cells in addition to tumor cells and have profound side-effects, CT antigens can be used for specific targeting of cancer cells using war-heads conjugated monoclonal antibodies or dendritic cell based immunotherapy [25]. Further, various studies have proposed the involvement of CT antigens in the regulation of transcription, cell proliferation and apoptosis [5]. Owing to their aberrant expression in various cancers, potent immunogenicity and limited or no expression in normal tissues other than germ cells in the testis, CT antigens are envisaged to be clinically important as biomarkers and therapeutic targets.

We earlier reported a novel testis specific gene, AKAP4 [14], encoded by X-chromosome that was shown to be involved in tethering PKA [26]. In the present study, we investigated the AKAP4 expression in various histotypes of breast carcinoma. We demonstrated that majority of breast cancer patients showed AKAP4 expression irrespective of clinical stages and histological grades of tumor suggesting that AKAP4 may have a potential role in disease progression. Till date, a total of 153 CT antigens have been reported, out of which 83 CT antigens are encoded by X-chromosome and are referred as CT-X antigens [27]. It is important to mention that earlier, very few studies have shown the CT-X antigen expression in breast cancer [27]. In this regard, an important CT-X antigen, melanoma associated antigen (MAGE) family demonstrated marked variations in expression of MAGE-A1, [6% (4 of 67)], MAGE-A2 [19% (13 of 67)], MAGE-A3 [10% (7 of 67)], MAGE-A4 [13% (9 of 67)], MAGE-A6 [15% (10 of 67)], and MAGE-A12 [9% (6 of 67)] in invasive breast cancer patients [28]. Similarly, New York-esophageal cancer-1 (NY-ESO-1) mRNA was shown to be expressed in 42% (22 of 37) breast cancer specimens [29]. In contrast, AKAP4 expression was observed in majority of breast cancer patients (85%) indicating its potential role in tumorigenesis.

In the past decade, gene expression profiling studies have led to the identification of various genes associated with cancer [30]. Although high throughput studies such as DNA microarray analysis and next generation sequencing reveal the encoded genetic alterations in cancer, these molecular markers are not implicated in clinical settings due to heterogeneity within tumors, high false-positives [30], and inter-assay discordances. It is noteworthy that, cancer is a complex disease involving alterations at the gene, RNA and protein level. Apart from associated genetic changes, cancer progression might also involve alterations in protein structure or function per se. Therefore, many of these differentially expressed genes are not promising candidate biomarkers due to discrepancy in gene and protein level. In this context, a well characterized CT antigen NY-ESO-1 mRNA was detected in 42% (37/88) breast cancer patients. It is important to mention that only 1 patient’s specimen showed NY-ESO-1 protein expression [29]. Therefore, it is very important to validate the protein expression of candidate gene biomarker under investigation. In our study, in situ RNA hybridization results revealed AKAP4 gene expression in 85% of total breast cancer patients. Further, our IHC studies demonstrated AKAP4 protein expression in all AKAP4 mRNA positive tissue specimens. We did not observe any discrepancy in AKAP4 gene or protein expression. Comparing with other CT antigens, MAGE-C1/CT7 expression was found only in 18% (22 of 124) of invasive breast cancer patients [31]. Recently, MAGE-A1, MAGE-A, and NY-ESO-1 protein expression was reported only in 33% (16/49), 33% (16/49), and 22% (11/49) of breast cancer patients, respectively by IHC analysis [32]. In addition, the three other CT antigens –GAGE, SAGE1 and Nuclear RNA export factor 2 (NXF2), were infrequently or rarely expressed, only in 3.5%, 2.2% and 1.8% of the breast cancer patients, respectively [33]. In contrast, AKAP4 showed expression at the gene and protein level in majority of breast cancer patients (85%) irrespective of histotypes, stages and clinical grades of breast cancer, suggestive of its potential as a biomarker and therapeutic candidate.

Identification of biomarker in the sera of cancer patients represent a better method for primary screening of breast cancer because of the minimal invasiveness associated with the procedure. Some of the breast cancer serum antigens such as CA 15-3 and CA 27.29 are elevated in less than 10% of early-disease patients [3]. Due to their low efficacy and sensitivity, no serum biomarkers are clinically validated for early diagnosis of breast cancer [3]. Antibodies against tumor associated antigens may be considered as more promising biomarkers than shedded tumor antigens for early diagnostic serum biomarkers. This is because, in early stage tumors, the shedding of tumor antigens is not very conspicuous; however the immune response is elevated in terms of detectable antibody titers. This is because of the clonal expansion of antibody response which can help in detection at the early stage of onset disease [34]. A recent study in primary breast cancer patients demonstrated auto-antibodies against a panel of tumor associated antigens including p53 (24%), c-myc (13%), BRCA1 (8%), BRCA2 (34%), and MUC1 (20%) by ELISA [35]. One of the well-studied non-X linked CT antigens; SPAG9 was shown to be highly immunogenic in 67% of epithelial ovarian cancer [6], 77% in renal cell carcinoma [7], 80% in breast cancer [8], 80% in cervical cancer [9], 78% in thyroid cancer [10], 88% in chronic myeloid leukemia [11], 70% in colorectal cancer [12] and 72% in endometrial cancer [13]. Most of the CT-X antigens studied so far are found to be poorly immunogenic in breast cancer [33]. In this regard, our investigation on CT-X antigen AKAP4 revealed that AKAP4 was immunogenic and generated humoral response in majority of breast cancer patients, as compared to healthy donors (P<0.001, Pearson’s Chi-square test). On the contrary, comparing with other well characterized X-linked CT antigen NY-ESO-1, auto antibodies against NY-ESO-1 were observed only in the sera of 1 of 62 breast cancer patients [29]. Yet another study demonstrated antibody response against Synaptonemal complex protein-1 (SCP-1) and Synovial sarcoma X break point 2 (SSX-2) only in 6% and 1% of breast cancer patients. However, no humoral response was observed against SSX-4, MAGE-3, MAGEC1 and MAGEC2 in breast cancer [36]. Further, recently in agreement with our observations, it was reported that AKAP4 was immunogenic in multiple myeloma (36%) [21] and prostate cancer patients (67%) [22]. Therefore, our findings of anti-AKAP4 antibodies in 79% of breast cancer patients are important from a clinical standpoint to develop novel assays for early diagnosis, and effective disease management.

Given consistencies in AKAP4 gene and protein expression, expression in various histotypes, different stages and grades of breast cancer, and humoral response against AKAP4 in breast cancer patients illustrates its potential diagnostic role as a biomarker in clinical settings. To the best of our knowledge, AKAP4 is the first X-linked CT antigen showing expression and humoral response in majority of breast cancer patients. The immune-privileged characteristic of testis and abundant expression of AKAP4 in breast cancer may provide the lead for further development of novel serum based tumor biomarker and might be a potential immunotherapeutic target. However, large scale clinical studies are warranted for exploring the implications of AKAP4 as a diagnostic biomarker.

### Conclusions

Early detection of breast cancer will help in diagnosis and effective treatment of breast cancer in its preinvasive state prior to metastasis. There are, at present, no existing validated tissue/serum biomarkers for breast cancer. We demonstrated AKAP4 expression (85%) and humoral response (79%) in breast cancer patients irrespective of their histotypes, grades and stages. Furthermore, anti-AKAP4 antibodies, present in the patient’s sera provides a basis for better, affordable and routine method of detection. AKAP4 expression in breast cancer in all clinicopathological stages and grades indicates its possible role in tumorigenesis and disease progression. Future large scale studies are warranted to explore its utility as an early diagnostic biomarker and immunotherapeutic target in breast cancer.

## Supporting Information

Figure S1 **AKAP4 expression and humoral response in different histotypes of breast cancer.** A, AKAP4 IRS in different histotypes, DCIS, IDC and IDC of breast cancer showed no significant difference among three sub-groups. B, anti-AKAP4 antibody titers in different histotypes of breast cancer showed no significant difference among various histotypes. Data is represented as mean±SE.
(TIF)

Figure S2 **Comparison of AKAP4 protein expression and circulating antibodies in different stages of breast carcinoma.** A, AKAP4 protein was found in breast cancer patients irrespective of their stages and showed no significant difference in clinical sub-groups. B, statistical analysis revealed no difference in circulating anti-AKAP4 antibody among various stages. Data is expressed as mean±SE.
(TIF)

Figure S3 **AKAP4 protein expression and humoral response in different histological grades of breast cancer.** A, AKAP4 protein expression was detected in all grades and showed no significant difference among various grades. B, anti-AKAP4 antibody titers were not significantly different among grades. Data is represented as mean±SE.
(TIF)
