# Supplementary figures and images for: A Novel Cancer Testis Antigen, A-Kinase Anchor Protein 4 (AKAP4) Is a Potential Biomarker for Breast Cancer
Source: PLoS One. 2013 Feb 22;8(2):e57095. doi: 10.1371/journal.pone.0057095 (PMC3579772; doi:10.1371/journal.pone.0057095)

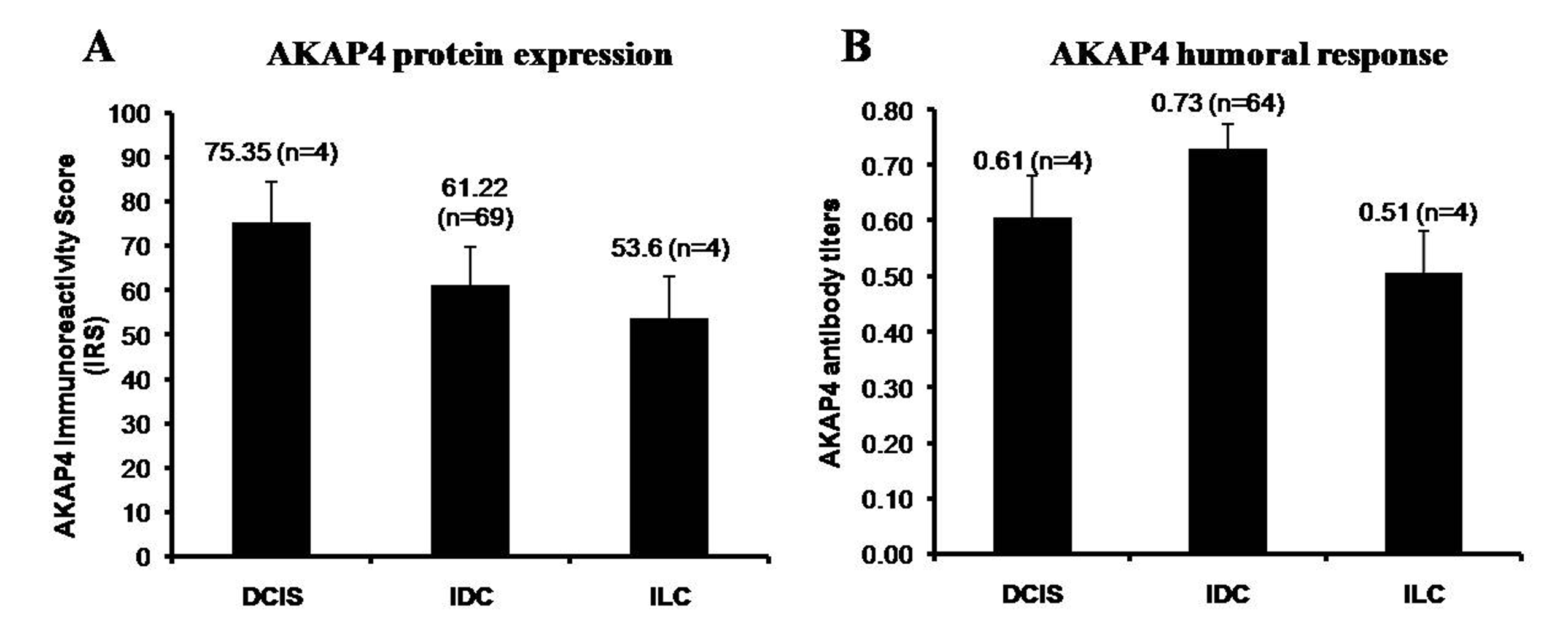

Supplement: Figure S1 — AKAP4 expression and humoral response in different histotypes of breast cancer. A, AKAP4 IRS in different histotypes, DCIS, IDC and IDC of breast cancer showed no significant difference among three sub-groups. B, anti-AKAP4 antibody titers in different histotypes of breast cancer showed no significant difference among various histotypes. Data is represented as mean±SE. (TIF) [file pone.0057095.s001.tif]

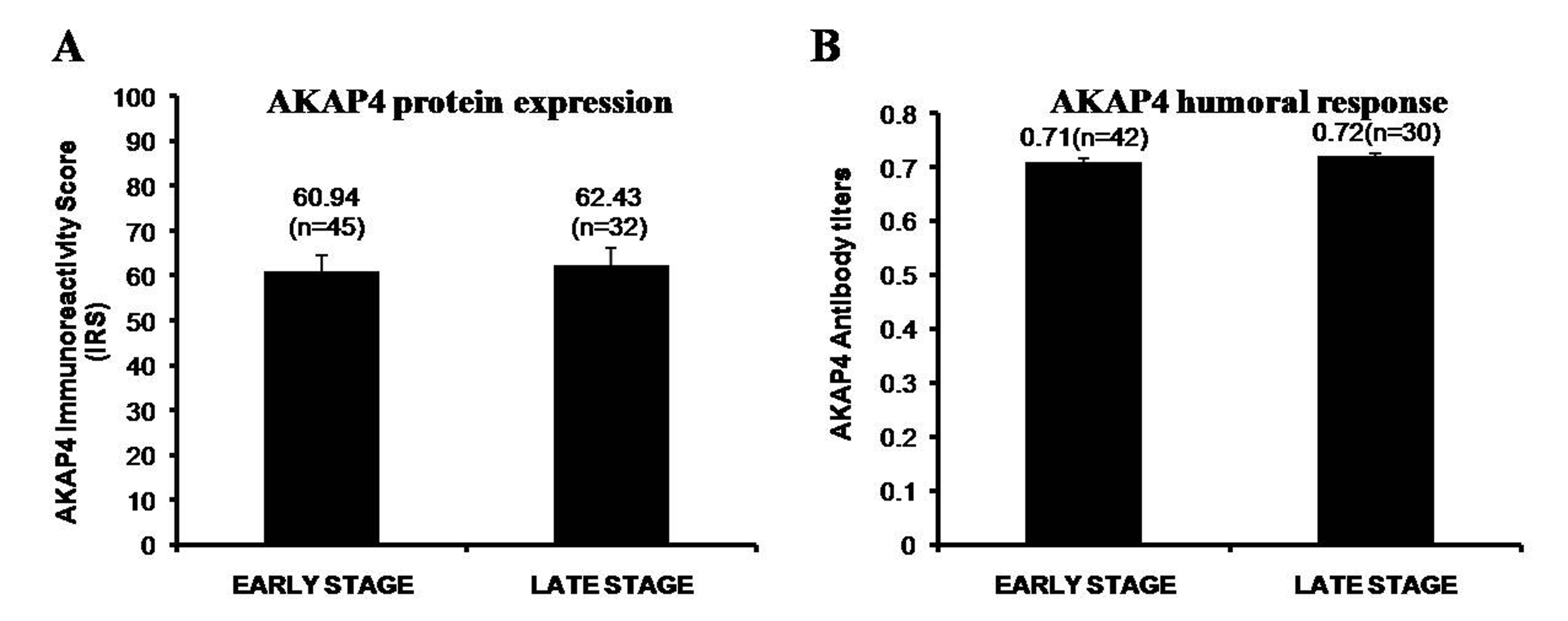

Supplement: Figure S2 — Comparison of AKAP4 protein expression and circulating antibodies in different stages of breast carcinoma. A, AKAP4 protein was found in breast cancer patients irrespective of their stages and showed no significant difference in clinical sub-groups. B, statistical analysis revealed no difference in circulating anti-AKAP4 antibody among various stages. Data is expressed as mean±SE. (TIF) [file pone.0057095.s002.tif]

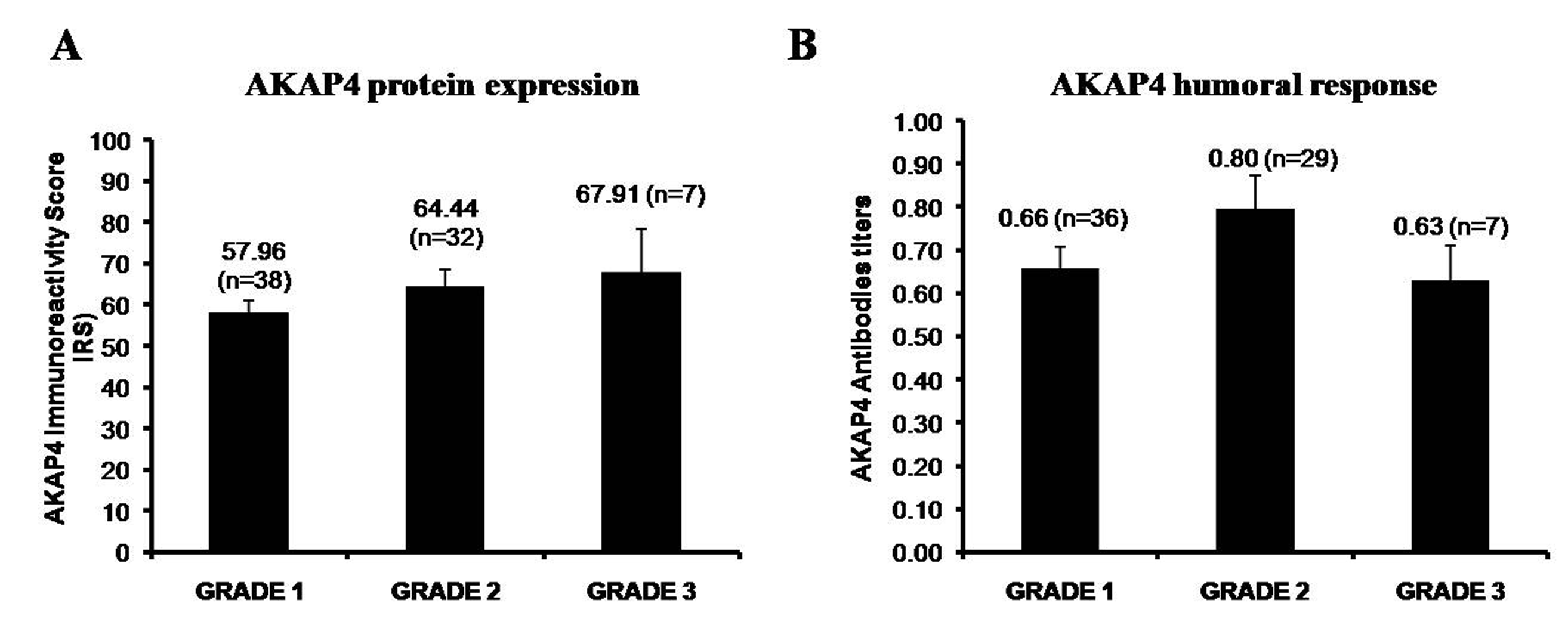

Supplement: Figure S3 — AKAP4 protein expression and humoral response in different histological grades of breast cancer. A, AKAP4 protein expression was detected in all grades and showed no significant difference among various grades. B, anti-AKAP4 antibody titers were not significantly different among grades. Data is represented as mean±SE. (TIF) [file pone.0057095.s003.tif]
